# {2,2′-[4-Methyl-4-aza­heptane-1,7-diylbis(nitrilo­methyl­idyne)]diphenolato}zinc(II)

**DOI:** 10.1107/S1600536808005060

**Published:** 2008-02-29

**Authors:** Xi-Shi Tai, Yi-Min Feng, Hua-Xiang Zhang

**Affiliations:** a Department of Chemistry, Weifang University, Weifang 261061, People’s Republic of China

## Abstract

In the title compound, [Zn(C_21_H_25_N_3_O_2_)], the Zn^II^ atom is five-coordinate from three N donor atoms and two O donor atoms of the dianion ligand in a distorted trigonal–bipyramidal arrangement. Three methyl­ene groups of the ligand are disordered over two orientations in a 0.555 (6):0.445 (6) ratio.

## Related literature

For related literature, see: Herzfeld & Nagy (1999 ▶); Niu *et al.* (2005 ▶).
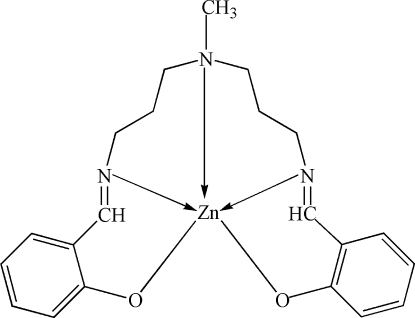


## Experimental

### 

#### Crystal data

[Zn(C_21_H_25_N_3_O_2_)]
*M* *_r_* = 416.81
Monoclinic, 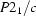

*a* = 6.7813 (6) Å
*b* = 13.9833 (12) Å
*c* = 20.766 (2) Å
β = 92.146 (1)°
*V* = 1967.7 (3) Å^3^
*Z* = 4
Mo *K*α radiation
μ = 1.27 mm^−1^
*T* = 298 (2) K
0.42 × 0.30 × 0.16 mm

#### Data collection

Bruker SMART CCD diffractometer
Absorption correction: multi-scan (*SADABS*; Bruker, 2000 ▶) *T*_min_ = 0.618, *T*_max_ = 0.823
9657 measured reflections
3465 independent reflections
2434 reflections with *I* > 2σ(*I*)
*R*_int_ = 0.031

#### Refinement

*R*[*F* ^2^ > 2σ(*F* ^2^)] = 0.039
*wR*(*F* ^2^) = 0.094
*S* = 1.04
3465 reflections
274 parameters
H-atom parameters constrained
Δρ_max_ = 0.31 e Å^−3^
Δρ_min_ = −0.23 e Å^−3^

### 

Data collection: *SMART* (Bruker, 2000 ▶); cell refinement: *SAINT* (Bruker, 2000 ▶); data reduction: *SAINT*; program(s) used to solve structure: *SHELXS97* (Sheldrick, 2008 ▶); program(s) used to refine structure: *SHELXL97* (Sheldrick, 2008 ▶); molecular graphics: *SHELXTL* (Sheldrick, 2008 ▶); software used to prepare material for publication: *SHELXTL*.

## Supplementary Material

Crystal structure: contains datablocks global, I. DOI: 10.1107/S1600536808005060/hb2702sup1.cif

Structure factors: contains datablocks I. DOI: 10.1107/S1600536808005060/hb2702Isup2.hkl

Additional supplementary materials:  crystallographic information; 3D view; checkCIF report

## Figures and Tables

**Table:** 

Zn1—O1	1.958 (2)
Zn1—O2	1.959 (2)
Zn1—N3	2.070 (3)
Zn1—N2	2.077 (3)
Zn1—N1	2.164 (3)

**Table:** 

N3—Zn1—N2	178.59 (12)

**Fig. 1.**
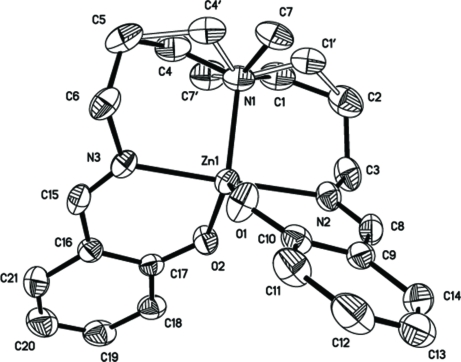
The molecular structure of (I) with 30% probabiility ellipsoids (H atoms and the minor disorder component omitted for clarity).

## supplementary crystallographic information

### Comment

Schiff-base ligands are able to coordinate to metal ions through their imine nitrogen atoms and another group, usually linked to the aldehyde moiety. They have long played a key role in coordination chemistry (*e.g.* Niu *et al.*, 2005; Herzfeld & Nagy, 1999). We now report the synthesis and structure of the title compound, (I).

The Zn^II^ center in (I) is five-coordinate with three N donor atoms and two O donor atoms of salicylaldehyde-*N*,*N*-bis-(3-aminopropyl)methylamine, and forms a distorted trigonal bipyramidal arrangement (Table 1, Fig. 1) with the O atoms in the equatorial sites. The dihedral angle between the aromatic rings is 72.23 (19)°.

### Experimental

1 mmol of Zn^II^ acetate was added to a solution of salicylaldehyde-*N*,*N*-bis(3-aminopropyl)methylamine (1 mmol) in 10 ml of ethanol. The mixture was continuously stirred for 3 h at refluxing temperature, evaporating some ethanol, then the product was collected by filtration, yield 68%. IR (KBr disk): 1614 (*m*) (C=N). Colourless blocks of (I) were grown by slow evaporation of an ethanol solution.

### Refinement

Three methylene groups (C1, C4, C7) of the ligand are disordered over two positions in a 0.555 (6):0.445 (6) ratio (sum of occupancies constrained to unity). The positions of all H atoms were fixed geometrically (C—H = 0.93–0.97 Å) and refined as riding with *U*_iso_(H) = 1.2*U*_eq_(C) or 1.5*U*_eq_(methyl C).

### Crystal data

**Table:**

[Zn(C_21_H_25_N_3_O_2_)]	*F*_000_ = 872
*M**_r_* = 416.81	*D*_x_ = 1.407 Mg m^−^^3^
Monoclinic, *P*2_1_/*c*	Mo *K*α radiation λ = 0.71073 Å
Hall symbol: -P 2ybc	Cell parameters from 3004 reflections
*a* = 6.7813 (6) Å	θ = 2.5–24.4º
*b* = 13.9833 (12) Å	µ = 1.27 mm^−^^1^
*c* = 20.766 (2) Å	*T* = 298 (2) K
β = 92.1460 (10)º	Block, colourless
*V* = 1967.7 (3) Å^3^	0.42 × 0.30 × 0.16 mm
*Z* = 4	

### Data collection

**Table:**

Bruker SMART CCD diffractometer	3465 independent reflections
Radiation source: fine-focus sealed tube	2434 reflections with *I* > 2σ(*I*)
Monochromator: graphite	*R*_int_ = 0.031
*T* = 298(2) K	θ_max_ = 25.0º
ω scans	θ_min_ = 1.8º
Absorption correction: multi-scan(SADABS; Bruker, 2000)	*h* = −4→8
*T*_min_ = 0.618, *T*_max_ = 0.823	*k* = −16→16
9657 measured reflections	*l* = −24→23

### Refinement

**Table:**

Refinement on *F*^2^	Secondary atom site location: difference Fourier map
Least-squares matrix: full	Hydrogen site location: inferred from neighbouring sites
*R*[*F*^2^ > 2σ(*F*^2^)] = 0.039	H-atom parameters constrained
*wR*(*F*^2^) = 0.094	*w* = 1/[σ^2^(*F*_o_^2^) + (0.036*P*)^2^ + 1.2726*P*] where *P* = (*F*_o_^2^ + 2*F*_c_^2^)/3
*S* = 1.04	(Δ/σ)_max_ = 0.001
3465 reflections	Δρ_max_ = 0.31 e Å^−^^3^
274 parameters	Δρ_min_ = −0.23 e Å^−^^3^
Primary atom site location: structure-invariant direct methods	Extinction correction: none

### Special details

**Table:**

Geometry. All e.s.d.'s (except the e.s.d. in the dihedral angle between two l.s. planes) are estimated using the full covariance matrix. The cell e.s.d.'s are taken into account individually in the estimation of e.s.d.'s in distances, angles and torsion angles; correlations between e.s.d.'s in cell parameters are only used when they are defined by crystal symmetry. An approximate (isotropic) treatment of cell e.s.d.'s is used for estimating e.s.d.'s involving l.s. planes.
Refinement. Refinement of *F*^2^ against ALL reflections. The weighted *R*-factor *wR* and goodness of fit *S* are based on *F*^2^, conventional *R*-factors *R* are based on *F*, with *F* set to zero for negative *F*^2^. The threshold expression of *F*^2^ > σ(*F*^2^) is used only for calculating *R*-factors(gt) *etc*. and is not relevant to the choice of reflections for refinement. *R*-factors based on *F*^2^ are statistically about twice as large as those based on *F*, and *R*- factors based on ALL data will be even larger.

### Fractional atomic coordinates and isotropic or equivalent isotropic displacement parameters (Å^2^)

**Table:**

	*x*	*y*	*z*	*U*_iso_*/*U*_eq_	Occ. (<1)
Zn1	0.05456 (5)	−0.00305 (3)	0.296700 (17)	0.04162 (13)	
N1	0.0566 (4)	0.1448 (2)	0.32765 (15)	0.0532 (7)	
N2	0.2021 (4)	−0.0409 (2)	0.38237 (13)	0.0497 (7)	
N3	−0.0914 (4)	0.0382 (2)	0.21187 (14)	0.0478 (7)	
O1	−0.1738 (3)	−0.07700 (18)	0.32321 (12)	0.0630 (7)	
O2	0.2801 (3)	−0.04353 (18)	0.24716 (10)	0.0547 (6)	
C1	0.2519 (12)	0.1720 (5)	0.3548 (4)	0.072 (3)	0.555 (6)
H1A	0.2479	0.2393	0.3660	0.087*	0.555 (6)
H1B	0.3468	0.1648	0.3214	0.087*	0.555 (6)
C1'	0.1189 (14)	0.1529 (6)	0.3952 (5)	0.068 (3)	0.445 (6)
H1'1	0.0253	0.1180	0.4205	0.081*	0.445 (6)
H1'2	0.1105	0.2197	0.4074	0.081*	0.445 (6)
C2	0.3289 (6)	0.1166 (3)	0.4143 (2)	0.0795 (13)	
H2A	0.4453	0.1485	0.4325	0.095*	0.555 (6)
H2B	0.2291	0.1169	0.4466	0.095*	0.555 (6)
H2'A	0.4218	0.1577	0.3931	0.095*	0.445 (6)
H2'B	0.3509	0.1257	0.4603	0.095*	0.445 (6)
C3	0.3807 (5)	0.0136 (3)	0.39799 (18)	0.0650 (11)	
H3A	0.4662	0.0126	0.3616	0.078*	
H3B	0.4512	−0.0156	0.4344	0.078*	
C4	0.0020 (11)	0.2101 (5)	0.2725 (4)	0.069 (2)	0.555 (6)
H4A	0.0941	0.2001	0.2385	0.083*	0.555 (6)
H4B	0.0163	0.2758	0.2870	0.083*	0.555 (6)
C4'	−0.1349 (14)	0.1873 (6)	0.3190 (5)	0.068 (3)	0.445 (6)
H4'1	−0.1318	0.2507	0.3379	0.081*	0.445 (6)
H4'2	−0.2292	0.1494	0.3420	0.081*	0.445 (6)
C5	−0.2105 (6)	0.1959 (3)	0.2442 (2)	0.0773 (13)	
H5A	−0.3016	0.2143	0.2769	0.093*	0.555 (6)
H5B	−0.2301	0.2398	0.2084	0.093*	0.555 (6)
H5'A	−0.3230	0.2385	0.2400	0.093*	0.445 (6)
H5'B	−0.1058	0.2214	0.2186	0.093*	0.445 (6)
C6	−0.2670 (5)	0.0966 (3)	0.22078 (19)	0.0621 (10)	
H6A	−0.3497	0.0662	0.2519	0.075*	
H6B	−0.3422	0.1014	0.1803	0.075*	
C7	−0.0990 (11)	0.1576 (5)	0.3790 (4)	0.069 (3)	0.555 (6)
H7A	−0.0995	0.2230	0.3931	0.104*	0.555 (6)
H7B	−0.0678	0.1166	0.4150	0.104*	0.555 (6)
H7C	−0.2269	0.1413	0.3608	0.104*	0.555 (6)
C7'	0.2059 (14)	0.1965 (6)	0.2876 (5)	0.073 (3)	0.445 (6)
H7'1	0.1720	0.1880	0.2427	0.109*	0.445 (6)
H7'2	0.3351	0.1709	0.2969	0.109*	0.445 (6)
H7'3	0.2049	0.2635	0.2979	0.109*	0.445 (6)
C8	0.1539 (5)	−0.1118 (3)	0.41727 (16)	0.0543 (9)	
H8	0.2420	−0.1287	0.4507	0.065*	
C9	−0.0230 (6)	−0.1678 (2)	0.40993 (16)	0.0523 (9)	
C10	−0.1786 (5)	−0.1465 (3)	0.36458 (16)	0.0496 (9)	
C11	−0.3492 (6)	−0.2041 (3)	0.36588 (19)	0.0689 (11)	
H11	−0.4561	−0.1905	0.3380	0.083*	
C12	−0.3599 (9)	−0.2800 (3)	0.4076 (2)	0.0901 (17)	
H12	−0.4735	−0.3173	0.4070	0.108*	
C13	−0.2084 (10)	−0.3019 (3)	0.4499 (2)	0.0916 (17)	
H13	−0.2180	−0.3540	0.4775	0.110*	
C14	−0.0421 (7)	−0.2467 (3)	0.45150 (17)	0.0714 (12)	
H14	0.0609	−0.2614	0.4807	0.086*	
C15	−0.0409 (5)	0.0154 (2)	0.15601 (17)	0.0539 (9)	
H15	−0.1270	0.0322	0.1220	0.065*	
C16	0.1365 (5)	−0.0338 (2)	0.13989 (16)	0.0466 (8)	
C17	0.2905 (5)	−0.0573 (2)	0.18560 (15)	0.0428 (8)	
C18	0.4635 (6)	−0.0977 (3)	0.16172 (18)	0.0579 (10)	
H18	0.5671	−0.1134	0.1904	0.070*	
C19	0.4829 (7)	−0.1144 (3)	0.0972 (2)	0.0728 (12)	
H19	0.6002	−0.1399	0.0830	0.087*	
C20	0.3324 (8)	−0.0942 (3)	0.0531 (2)	0.0824 (14)	
H20	0.3459	−0.1070	0.0095	0.099*	
C21	0.1631 (7)	−0.0552 (3)	0.07466 (18)	0.0714 (12)	
H21	0.0606	−0.0421	0.0449	0.086*	

### Atomic displacement parameters (Å^2^)

**Table:**

	*U*^11^	*U*^22^	*U*^33^	*U*^12^	*U*^13^	*U*^23^
Zn1	0.0380 (2)	0.0434 (2)	0.0434 (2)	−0.00014 (19)	0.00068 (15)	0.00249 (18)
N1	0.0469 (18)	0.0445 (17)	0.069 (2)	−0.0050 (14)	0.0094 (15)	−0.0073 (15)
N2	0.0418 (16)	0.0634 (18)	0.0437 (16)	0.0007 (14)	−0.0021 (13)	−0.0026 (15)
N3	0.0404 (16)	0.0486 (16)	0.0541 (18)	0.0054 (13)	−0.0037 (14)	0.0089 (13)
O1	0.0432 (14)	0.0714 (18)	0.0737 (16)	−0.0154 (13)	−0.0062 (12)	0.0271 (14)
O2	0.0399 (13)	0.0782 (17)	0.0459 (14)	0.0156 (12)	−0.0010 (11)	−0.0063 (12)
C1	0.068 (5)	0.053 (5)	0.096 (7)	−0.012 (4)	0.009 (5)	−0.013 (4)
C1'	0.069 (7)	0.054 (6)	0.081 (7)	0.002 (5)	0.008 (6)	−0.013 (5)
C2	0.063 (3)	0.089 (3)	0.086 (3)	−0.006 (2)	−0.008 (2)	−0.029 (3)
C3	0.046 (2)	0.093 (3)	0.055 (2)	−0.005 (2)	−0.0089 (17)	−0.006 (2)
C4	0.070 (6)	0.047 (4)	0.091 (6)	−0.006 (4)	0.013 (5)	−0.001 (4)
C4'	0.064 (6)	0.050 (5)	0.090 (8)	0.004 (5)	0.010 (6)	−0.009 (5)
C5	0.065 (3)	0.061 (3)	0.106 (4)	0.022 (2)	−0.001 (3)	0.010 (2)
C6	0.044 (2)	0.068 (3)	0.074 (3)	0.0147 (19)	−0.0040 (19)	0.015 (2)
C7	0.060 (5)	0.071 (5)	0.078 (6)	0.003 (4)	0.021 (4)	−0.025 (4)
C7'	0.070 (7)	0.051 (5)	0.098 (8)	−0.019 (5)	0.019 (6)	0.005 (5)
C8	0.055 (2)	0.071 (3)	0.0370 (19)	0.015 (2)	−0.0002 (17)	0.0036 (18)
C9	0.070 (3)	0.046 (2)	0.0424 (19)	0.0061 (19)	0.0162 (19)	0.0017 (16)
C10	0.053 (2)	0.050 (2)	0.047 (2)	−0.0071 (18)	0.0126 (18)	−0.0048 (17)
C11	0.073 (3)	0.071 (3)	0.063 (2)	−0.026 (2)	0.013 (2)	−0.008 (2)
C12	0.134 (5)	0.071 (3)	0.068 (3)	−0.051 (3)	0.038 (3)	−0.014 (2)
C13	0.166 (6)	0.054 (3)	0.057 (3)	−0.021 (3)	0.040 (3)	0.001 (2)
C14	0.108 (4)	0.059 (3)	0.048 (2)	0.011 (3)	0.020 (2)	0.0062 (19)
C15	0.055 (2)	0.056 (2)	0.049 (2)	0.0014 (18)	−0.0116 (17)	0.0142 (17)
C16	0.055 (2)	0.0400 (18)	0.0454 (19)	−0.0023 (16)	0.0033 (17)	0.0027 (15)
C17	0.046 (2)	0.0357 (18)	0.047 (2)	0.0007 (15)	0.0044 (16)	0.0007 (15)
C18	0.058 (2)	0.052 (2)	0.064 (2)	0.0077 (19)	0.0106 (19)	−0.0007 (18)
C19	0.084 (3)	0.062 (3)	0.075 (3)	0.012 (2)	0.032 (3)	−0.003 (2)
C20	0.118 (4)	0.076 (3)	0.055 (3)	0.005 (3)	0.026 (3)	−0.005 (2)
C21	0.098 (3)	0.070 (3)	0.046 (2)	0.003 (3)	−0.002 (2)	0.009 (2)

### Geometric parameters (Å, °)

**Table:**

Zn1—O1	1.958 (2)	C5—H5A	0.9700
Zn1—O2	1.959 (2)	C5—H5B	0.9700
Zn1—N3	2.070 (3)	C5—H5'A	0.9700
Zn1—N2	2.077 (3)	C5—H5'B	0.9700
Zn1—N1	2.164 (3)	C6—H6A	0.9700
N1—C4'	1.434 (9)	C6—H6B	0.9700
N1—C1'	1.454 (9)	C7—H7A	0.9600
N1—C1	1.470 (8)	C7—H7B	0.9600
N1—C4	1.501 (8)	C7—H7C	0.9600
N1—C7'	1.518 (9)	C7'—H7'1	0.9600
N1—C7	1.539 (7)	C7'—H7'2	0.9600
N2—C8	1.278 (4)	C7'—H7'3	0.9600
N2—C3	1.457 (4)	C8—C9	1.436 (5)
N3—C15	1.262 (4)	C8—H8	0.9300
N3—C6	1.461 (4)	C9—C14	1.409 (5)
O1—C10	1.298 (4)	C9—C10	1.419 (5)
O2—C17	1.297 (4)	C10—C11	1.411 (5)
C1—C2	1.532 (9)	C11—C12	1.374 (6)
C1—H1A	0.9700	C11—H11	0.9300
C1—H1B	0.9700	C12—C13	1.361 (7)
C1'—C2	1.550 (10)	C12—H12	0.9300
C1'—H1'1	0.9700	C13—C14	1.367 (6)
C1'—H1'2	0.9700	C13—H13	0.9300
C2—C3	1.525 (6)	C14—H14	0.9300
C2—H2A	0.9700	C15—C16	1.436 (5)
C2—H2B	0.9700	C15—H15	0.9300
C2—H2'B	0.9700	C16—C21	1.405 (5)
C3—H3A	0.9700	C16—C17	1.423 (4)
C3—H3B	0.9700	C17—C18	1.409 (4)
C4—C5	1.549 (8)	C18—C19	1.370 (5)
C4—H4A	0.9700	C18—H18	0.9300
C4—H4B	0.9700	C19—C20	1.375 (6)
C4'—C5	1.621 (11)	C19—H19	0.9300
C4'—H4'1	0.9700	C20—C21	1.362 (6)
C4'—H4'2	0.9700	C20—H20	0.9300
C5—C6	1.515 (5)	C21—H21	0.9300
			
O1—Zn1—O2	129.52 (11)	N1—C4—H5'B	146.2
O1—Zn1—N3	91.62 (10)	C5—C4—H5'B	38.5
O2—Zn1—N3	89.51 (10)	H4A—C4—H5'B	75.7
O1—Zn1—N2	89.15 (11)	H4B—C4—H5'B	101.3
O2—Zn1—N2	90.90 (10)	N1—C4'—C5	113.8 (6)
N3—Zn1—N2	178.59 (12)	N1—C4'—H4'1	108.8
O1—Zn1—N1	114.66 (11)	C5—C4'—H4'1	108.8
O2—Zn1—N1	115.81 (11)	N1—C4'—H4'2	108.8
N3—Zn1—N1	89.10 (12)	C5—C4'—H4'2	108.8
N2—Zn1—N1	89.51 (12)	H4'1—C4'—H4'2	107.7
C4'—N1—C1'	108.5 (6)	C6—C5—C4	117.4 (4)
C4'—N1—C1	137.7 (5)	C6—C5—C4'	107.9 (4)
C1'—N1—C1	52.0 (5)	C4—C5—C4'	52.4 (4)
C4'—N1—C4	57.0 (5)	C6—C5—H5A	108.0
C1'—N1—C4	138.0 (5)	C4—C5—H5A	108.0
C1—N1—C4	109.2 (5)	C4'—C5—H5A	62.3
C4'—N1—C7'	110.7 (6)	C6—C5—H5B	108.0
C1'—N1—C7'	108.4 (6)	C4—C5—H5B	108.0
C1—N1—C7'	58.5 (5)	C4'—C5—H5B	144.1
C4—N1—C7'	56.1 (5)	H5A—C5—H5B	107.2
C4'—N1—C7	52.9 (5)	C6—C5—H5'A	110.2
C1'—N1—C7	60.3 (5)	C4—C5—H5'A	132.3
C1—N1—C7	109.6 (5)	C4'—C5—H5'A	110.6
C4—N1—C7	107.5 (5)	H5A—C5—H5'A	51.7
C7'—N1—C7	144.4 (5)	H5B—C5—H5'A	57.0
C4'—N1—Zn1	111.3 (4)	C6—C5—H5'B	110.0
C1'—N1—Zn1	111.1 (4)	C4—C5—H5'B	58.0
C1—N1—Zn1	110.9 (3)	C4'—C5—H5'B	109.9
C4—N1—Zn1	110.9 (3)	H5A—C5—H5'B	141.6
C7'—N1—Zn1	106.9 (4)	H5B—C5—H5'B	55.2
C7—N1—Zn1	108.7 (3)	H5'A—C5—H5'B	108.3
C8—N2—C3	120.5 (3)	N3—C6—C5	110.8 (3)
C8—N2—Zn1	124.0 (2)	N3—C6—H6A	109.5
C3—N2—Zn1	115.3 (2)	C5—C6—H6A	109.5
C15—N3—C6	120.5 (3)	N3—C6—H6B	109.5
C15—N3—Zn1	125.0 (2)	C5—C6—H6B	109.5
C6—N3—Zn1	114.4 (2)	H6A—C6—H6B	108.1
C10—O1—Zn1	128.4 (2)	N1—C7—H7A	109.5
C17—O2—Zn1	129.2 (2)	N1—C7—H7B	109.5
N1—C1—C2	117.0 (6)	N1—C7—H7C	109.5
N1—C1—H1A	108.1	N1—C7'—H7'1	109.5
C2—C1—H1A	108.1	N1—C7'—H7'2	109.5
N1—C1—H1B	108.1	H7'1—C7'—H7'2	109.5
C2—C1—H1B	108.1	N1—C7'—H7'3	109.5
H1A—C1—H1B	107.3	H7'1—C7'—H7'3	109.5
N1—C1—H2'A	153.9	H7'2—C7'—H7'3	109.5
C2—C1—H2'A	38.4	N2—C8—C9	126.4 (3)
H1A—C1—H2'A	91.9	N2—C8—H8	116.8
H1B—C1—H2'A	80.6	C9—C8—H8	116.8
N1—C1'—C2	116.9 (7)	C14—C9—C10	119.2 (4)
N1—C1'—H1'1	108.1	C14—C9—C8	117.2 (4)
C2—C1'—H1'1	108.1	C10—C9—C8	123.6 (3)
N1—C1'—H1'2	108.1	O1—C10—C11	118.8 (3)
C2—C1'—H1'2	108.1	O1—C10—C9	124.0 (3)
H1'1—C1'—H1'2	107.3	C11—C10—C9	117.2 (3)
C3—C2—C1	111.9 (4)	C12—C11—C10	121.1 (4)
C3—C2—C1'	118.0 (4)	C12—C11—H11	119.5
C1—C2—C1'	49.2 (4)	C10—C11—H11	119.5
C3—C2—H2A	109.2	C13—C12—C11	121.6 (4)
C1—C2—H2A	109.2	C13—C12—H12	119.2
C1'—C2—H2A	132.5	C11—C12—H12	119.2
C3—C2—H2B	109.2	C12—C13—C14	119.4 (4)
C1—C2—H2B	109.2	C12—C13—H13	120.3
C1'—C2—H2B	61.1	C14—C13—H13	120.3
H2A—C2—H2B	107.9	C13—C14—C9	121.5 (4)
C3—C2—H2'A	107.5	C13—C14—H14	119.3
C1—C2—H2'A	62.8	C9—C14—H14	119.3
C1'—C2—H2'A	107.2	N3—C15—C16	126.4 (3)
H2A—C2—H2'A	51.1	N3—C15—H15	116.8
H2B—C2—H2'A	142.4	C16—C15—H15	116.8
C3—C2—H2'B	108.3	C21—C16—C17	118.5 (3)
C1—C2—H2'B	139.8	C21—C16—C15	117.7 (3)
C1'—C2—H2'B	108.4	C17—C16—C15	123.7 (3)
H2A—C2—H2'B	57.6	O2—C17—C18	118.9 (3)
H2B—C2—H2'B	53.4	O2—C17—C16	123.9 (3)
H2'A—C2—H2'B	107.0	C18—C17—C16	117.2 (3)
N2—C3—C2	110.3 (3)	C19—C18—C17	121.6 (4)
N2—C3—H3A	109.6	C19—C18—H18	119.2
C2—C3—H3A	109.6	C17—C18—H18	119.2
N2—C3—H3B	109.6	C18—C19—C20	121.4 (4)
C2—C3—H3B	109.6	C18—C19—H19	119.3
H3A—C3—H3B	108.1	C20—C19—H19	119.3
N1—C4—C5	114.2 (5)	C21—C20—C19	118.5 (4)
N1—C4—H4A	108.7	C21—C20—H20	120.8
C5—C4—H4A	108.7	C19—C20—H20	120.8
N1—C4—H4B	108.7	C20—C21—C16	122.8 (4)
C5—C4—H4B	108.7	C20—C21—H21	118.6
H4A—C4—H4B	107.6	C16—C21—H21	118.6
			
O1—Zn1—N1—C4'	−43.0 (5)	N1—C1—C2—C1'	−39.1 (6)
O2—Zn1—N1—C4'	137.4 (5)	N1—C1'—C2—C3	−56.1 (9)
N3—Zn1—N1—C4'	48.4 (5)	N1—C1'—C2—C1	39.6 (6)
N2—Zn1—N1—C4'	−131.8 (5)	C8—N2—C3—C2	−116.9 (4)
O1—Zn1—N1—C1'	78.0 (5)	Zn1—N2—C3—C2	68.8 (4)
O2—Zn1—N1—C1'	−101.6 (5)	C1—C2—C3—N2	−69.8 (5)
N3—Zn1—N1—C1'	169.4 (5)	C1'—C2—C3—N2	−15.6 (7)
N2—Zn1—N1—C1'	−10.8 (5)	C4'—N1—C4—C5	−38.7 (6)
O1—Zn1—N1—C1	134.0 (4)	C1'—N1—C4—C5	−119.8 (8)
O2—Zn1—N1—C1	−45.6 (4)	C1—N1—C4—C5	−173.8 (5)
N3—Zn1—N1—C1	−134.6 (4)	C7'—N1—C4—C5	160.4 (8)
N2—Zn1—N1—C1	45.2 (4)	C7—N1—C4—C5	−55.0 (6)
O1—Zn1—N1—C4	−104.5 (4)	Zn1—N1—C4—C5	63.7 (6)
O2—Zn1—N1—C4	75.9 (4)	C1'—N1—C4'—C5	172.3 (6)
N3—Zn1—N1—C4	−13.1 (4)	C1—N1—C4'—C5	119.0 (8)
N2—Zn1—N1—C4	166.7 (4)	C4—N1—C4'—C5	36.6 (5)
O1—Zn1—N1—C7'	−163.9 (5)	C7'—N1—C4'—C5	53.5 (8)
O2—Zn1—N1—C7'	16.5 (5)	C7—N1—C4'—C5	−163.0 (9)
N3—Zn1—N1—C7'	−72.5 (5)	Zn1—N1—C4'—C5	−65.2 (7)
N2—Zn1—N1—C7'	107.3 (5)	N1—C4—C5—C6	−56.3 (7)
O1—Zn1—N1—C7	13.5 (4)	N1—C4—C5—C4'	35.9 (5)
O2—Zn1—N1—C7	−166.1 (4)	N1—C4'—C5—C6	73.6 (7)
N3—Zn1—N1—C7	104.9 (4)	N1—C4'—C5—C4	−37.6 (5)
N2—Zn1—N1—C7	−75.3 (4)	C15—N3—C6—C5	−109.7 (4)
O1—Zn1—N2—C8	19.3 (3)	Zn1—N3—C6—C5	71.3 (3)
O2—Zn1—N2—C8	−110.2 (3)	C4—C5—C6—N3	−16.8 (6)
N3—Zn1—N2—C8	143 (4)	C4'—C5—C6—N3	−73.1 (5)
N1—Zn1—N2—C8	134.0 (3)	C3—N2—C8—C9	175.9 (3)
O1—Zn1—N2—C3	−166.6 (2)	Zn1—N2—C8—C9	−10.3 (5)
O2—Zn1—N2—C3	63.9 (2)	N2—C8—C9—C14	176.4 (3)
N3—Zn1—N2—C3	−43 (4)	N2—C8—C9—C10	−5.1 (6)
N1—Zn1—N2—C3	−51.9 (2)	Zn1—O1—C10—C11	−165.7 (3)
O1—Zn1—N3—C15	−115.6 (3)	Zn1—O1—C10—C9	15.4 (5)
O2—Zn1—N3—C15	13.9 (3)	C14—C9—C10—O1	−178.3 (3)
N2—Zn1—N3—C15	121 (4)	C8—C9—C10—O1	3.2 (5)
N1—Zn1—N3—C15	129.7 (3)	C14—C9—C10—C11	2.7 (5)
O1—Zn1—N3—C6	63.3 (2)	C8—C9—C10—C11	−175.8 (3)
O2—Zn1—N3—C6	−167.2 (2)	O1—C10—C11—C12	178.4 (4)
N2—Zn1—N3—C6	−60 (4)	C9—C10—C11—C12	−2.6 (5)
N1—Zn1—N3—C6	−51.3 (2)	C10—C11—C12—C13	0.9 (7)
O2—Zn1—O1—C10	68.2 (3)	C11—C12—C13—C14	0.7 (7)
N3—Zn1—O1—C10	158.9 (3)	C12—C13—C14—C9	−0.5 (6)
N2—Zn1—O1—C10	−22.3 (3)	C10—C9—C14—C13	−1.2 (5)
N1—Zn1—O1—C10	−111.3 (3)	C8—C9—C14—C13	177.3 (4)
O1—Zn1—O2—C17	77.3 (3)	C6—N3—C15—C16	173.9 (3)
N3—Zn1—O2—C17	−14.4 (3)	Zn1—N3—C15—C16	−7.2 (5)
N2—Zn1—O2—C17	166.9 (3)	N3—C15—C16—C21	178.1 (4)
N1—Zn1—O2—C17	−103.2 (3)	N3—C15—C16—C17	−5.4 (6)
C4'—N1—C1—C2	115.1 (9)	Zn1—O2—C17—C18	−172.4 (2)
C1'—N1—C1—C2	40.2 (6)	Zn1—O2—C17—C16	7.7 (5)
C4—N1—C1—C2	176.8 (5)	C21—C16—C17—O2	−177.9 (3)
C7'—N1—C1—C2	−158.2 (8)	C15—C16—C17—O2	5.6 (5)
C7—N1—C1—C2	59.2 (7)	C21—C16—C17—C18	2.2 (5)
Zn1—N1—C1—C2	−60.8 (6)	C15—C16—C17—C18	−174.2 (3)
C4'—N1—C1'—C2	−176.4 (6)	O2—C17—C18—C19	179.7 (3)
C1—N1—C1'—C2	−39.6 (6)	C16—C17—C18—C19	−0.5 (5)
C4—N1—C1'—C2	−115.5 (8)	C17—C18—C19—C20	−1.4 (6)
C7'—N1—C1'—C2	−56.1 (8)	C18—C19—C20—C21	1.3 (7)
C7—N1—C1'—C2	161.1 (9)	C19—C20—C21—C16	0.6 (7)
Zn1—N1—C1'—C2	61.0 (7)	C17—C16—C21—C20	−2.4 (6)
N1—C1—C2—C3	69.7 (7)	C15—C16—C21—C20	174.3 (4)
